# Aldosterone regulates a 5ʹ variant sgk1 transcript via a shared hormone response element in the sgk1 5ʹ regulatory region

**DOI:** 10.14814/phy2.13221

**Published:** 2017-04-13

**Authors:** Nandita S. Raikwar, Christie P. Thomas

**Affiliations:** ^1^ Department of Internal MedicineUniversity of Iowa College of MedicineIowa CityIowa; ^2^ The Graduate Program in Molecular BiologyUniversity of Iowa College of MedicineIowa CityIowa; ^3^ The Veterans Affairs Medical CenterIowa CityIowa

**Keywords:** Aldosterone, epithelial Na^+^ channel, Na^+^ transport

## Abstract

We previously identified a 5ʹ variant alternate transcript of Sgk1 (Sgk1_v3) encoding an NH_2_‐terminal variant Sgk1 isoform, Sgk1_i3 that, like Sgk1, is expressed in the distal convoluted tubule, connecting tubule and collecting duct and can stimulate epithelial Na^+^ transport (Am J Physiol Renal Physiol 303: F1527–F1533, 2012). We now demonstrate that, similar to Sgk1, aldosterone and glucocorticoids stimulate Sgk1_v3 expression in cell lines from the collecting duct and airway epithelia. In mice, short term aldosterone infusion and maneuvers that increase endogenous aldosterone secretion including dietary Na^+^ deprivation and K^+^ loading increases distal nephron Sgk1_v3 expression in vivo. Although Sgk1_v3 has a different 5ʹ proximal regulatory region from Sgk1, the transcription start sites are less than 1000 bp apart. We cloned the 5ʹ regulatory region for Sgk1 and Sgk_v3 upstream of a luciferase gene and by deletion and reporter gene analysis we localized the corticosteroid regulatory region for Sgk1_v3 to a glucocorticoid response element (GRE) that had previously been identified for Sgk1 (Am J Physiol Endo Metab 283: E971–E979, 2002). We tested this element with MR in an MR‐null cell line and demonstrate that aldosterone stimulates Sgk1 and Sgk1_v3 via this GRE. We conclude that corticosteroids stimulate Sgk1 and Sgk1_v3 expression in epithelial cells via activation of a common conserved GRE in the 5ʹ flanking region of Sgk1.

## Introduction

Serum and glucocorticoid regulated kinase 1 (Sgk1) is a ubiquitiously expressed kinase that regulates a number of transport processes particularly in the kidney and brain (Lang et al. 2010; Pao 2012). Sgk1 in turn is regulated by a large number of hormones including corticosteroids, gonadotropins, erythropoietin, cytokines, and growth factors (Lang et al. 2014). Sgk1 is an unstable protein that is activated by phosphorylation and is stimulated in response to a number of cell stressors including heat shock, cell shrinkage, oxidative stress, and UV radiation. Multiple lysines in the NH_2_ terminus of Sgk1 are rapidly ubiquitinated which targets Sgk1 to the proteasome (Brickley et al. 2002).

One of the principal targets of Sgk1 action is the epithelial sodium channel (ENaC) which regulates Na^+^ reabsorption and K^+^ secretion in the 2nd part of the distal convoluted tubule (DCT2), the connecting tubule (CNT) and the collecting duct (CCD) (Ellison et al. 2016). Sgk1 regulates ENaC in multiple ways including an increase in transcription of αENaC, a reduction in retrieval and degradation of the cell surface expressed ENaC complex and an increase in the channelʹs open probability (Debonneville et al. 2001; Snyder et al. 2002; Diakov and Korbmacher 2004; Zhang et al. 2007). Sgk1 is transcriptionally regulated by glucocorticoids via a glucocorticoid response element (GRE) in its 5ʹ flanking region (Webster et al. 1993; Itani et al. 2002). The relevance of Sgk1 in the regulation of Na^+^ and K^+^ homeostasis is demonstrated in both constitutive and inducible Sgk1 knockout (KO) mice where increased urinary Na^+^ wasting, low blood pressure and impaired K^+^ excretion are evident (Wulff et al. 2002; Faresse et al. 2012; Al‐Qusairi et al. 2016).

Others and we have previously reported alternate 5ʹ transcripts of Sgk1 that are translated to NH_2_ terminal variant isoforms. Sgk1_v2 is widely expressed but, in contrast to the originally reported principal Sgk1 transcript, is not regulated by glucocorticoids (Raikwar et al. 2008). Sgk1_v3 is expressed in a number of epithelial cells that have amiloride‐sensitive Na^+^ transport including the DCT, CNT and CCD and this transcript is regulated by aldosterone in collecting duct epithelia in culture (Raikwar et al. 2012). Both encoded isoforms, Sgk1_i2 and Sgk1_i3, have different NH_2_ termini, are less susceptible to ubiquitination and appear to be more stable than Sgk1 protein. Sgk1_i2 is targeted to the plasma membrane and stimulates a number of cation channels of the ENaC/degenerin family while Sgk1_i3 is more abundantly and widely expressed and is also a potent stimulator of ENaC‐mediated Na^+^ transport (Arteaga et al. 2008; Raikwar et al. 2008, 2012).

In this manuscript we show that short‐term aldosterone infusion and maneuvers that increase endogenous aldosterone secretion including dietary Na^+^ deprivation and K^+^ loading increases distal nephron Sgk1_v3 expression in vivo. We demonstrate that corticosteroids stimulate Sgk1 and Sgk1_v3 expression in epithelial cells via activation of a common conserved GRE in the 5ʹ flanking region of Sgk1.

## Material and Methods

### Materials

Aldosterone, amiloride, dexamethasone, insulin, selenium, transferrin, triiodothyronine, EGF, puromycin, and D‐Glucose were purchased from Sigma‐Aldrich (St. Louis, MO). Rat tail collagen was obtained from BD Biosciences (San Jose, CA). All cell culture media were obtained from Life Technologies (Gaithersburg, MD). The reporter vectors, pGL3basic, pGL4.1, and pRL‐SV40 were obtained from Promega (Madison, WI) and GIPZ shRNA Lentiviral particles were purchased from GE Dharmacon (Lafayette, CO).

### Cell culture, electrophysiology, and generation of stable Sgk1 shRNA expressing cells

Human embryonic kidney cell line HEK293 was cultured in high glucose DMEM containing 10% FBS and 1% penicillin‐streptomycin. MpkCCD_c14_ cells were cultured in defined media containing 2% FBS, antibiotics and other hormones, as previously described (Bens et al. 1999; Itani et al. 2005). A549 and H441 human lung epithelial cells and HT‐29 colonic epithelial cells were cultured as previously described (Mick et al. 2001; Itani et al. 2002).

MpkCCD_c14_ cells were transduced with each of three different GIPZ shRNAmir lentiviral particles specific to Sgk1 (clone ID: V3LHS_328980, V3LHS_636735, V3LHS_636738) or with non‐silencing shRNAmir control lentivirus according to manufacturer's instructions. Cells were maintained under puromycin selection for isolation of single cell clones. Individual clones were expanded to generate stable cell lines expressing shRNAs. Wild type mpkCCD_c14_ cells or stable mpkCCD_c14_ GIPZ lentiviral clones were seeded on collagen‐coated transwell filters (12‐mm‐diameter Millicell‐PCF, Millipore) and short‐circuit measurements (*I*_sc_) were measured in culture media at 37°C in Ussing chambers as described previously (Raikwar et al. 2012).

### Animals

7–8 weeks old female C57BL/6 mice were purchased from Jackson Laboratory (Bar Harbor, ME). All animals were fed regular rodent chow until start of experiments. Animals were then fed 0.1% NaCl (TD 92238) or 8% NaCl (TD 92012, Harlan Teklad) for 1 week or a 10% K diet (high K) or 0.1% K diet (Low K, a gift from Wenhui Wang, Valhalla, NY) for 1 week before sacrifice. In other experiments, aldosterone (1.5 mg/kg body weight) or vehicle diluted in PBS was injected intraperitoneally 2 h prior to sacrifice. All experiments were performed following protocols that were approved by the VAMC Institutional Animal Care and Use Committee.

### Plasmid constructs

The 5ʹ flanking region of Sgk1 upstream of exon 1 has been cloned previously into the reporter vector, pG3basic (Itani et al. 2002). A 2579 bp *Spe1‐HindIII* piece of this 5ʹ sequence that encompassed 1558 bp of sequence 5ʹ to exon 1d and the remaining sequence downstream including 117 bp of exon 1 was subcloned upstream of a modified firefly (*Photinus Pyralis)* luciferase in pGL4.1 to give Sgk1(1 + 3)luciferase. Then ~1000 bp downstream of exon 1d was removed by *NcoI* digestion to give Sgk1(3)luciferase which included 1558 bp of sequence 5ʹ and flanking exon 1d and 58 bp of exon 1d. The 5ʹ flanking sequence upstream of exon 1d including a previously mutated GRE was similarly subcloned into pGL4.1 to give Sgk1(3)mutGRE. Separately, the initial ~1900 bp of 5ʹ sequence in Sgk1(1 + 3)luciferase was removed by StuI and Acc65I digestion to give Sgk1(1)luciferase which included 532 bp 5ʹ and flanking exon 1.

### Transient transfection and analysis of reporter activity

A549 and HT‐29 cells grown in 24‐well plates were transfected using LipofectAMINE 2000 from Life Technologies (Grand Island, NY), with 1 *μ*g of the promoter‐reporter construct and as a control for transfection efficiency, 0.5 *μ*g of pRL‐SV40 (Promega), a plasmid vector where the SV40 viral promoter drives sea pansy luciferase (*Renilla reniformis*). In HT‐29 experiments, 0.5 *μ*g of a rat MR expression vector (gift from David Pearce) or an empty plasmid, pcDNA3 (Life Technologies,), was cotransfected with the luciferase plasmids. In some experiments, the αENaC promoter‐luciferase construct (‐141 + 55) that does not contain a functional GRE was used as a negative control and compared to a construct containing two copies of the Sgk1 GRE at ‐142nt, *α*ENaC_Sgk1_2xGREluciferase (Itani et al. 2002). The day after transfection, cells were treated with 100 nmol/L dexamethasone or aldosterone or vehicle and 24 h later cell lysates were prepared and reporter gene activity were performed with the Dual Luciferase Assay Kit (Promega) as previously described (Mick et al. 2001).

### Total RNA isolation and cDNA preparation

Total RNA from cultured cells was prepared with AbsolutelyRNA miniprep kit while total RNA from microdissected distal nephron segments was extracted with Absolutely RNA nanoprep kit (both from Agilent Technologies, La Jolla, CA). Equal amounts of RNA were subjected to reverse transcription with SuperScript^®^ VILO™ (Life Technologies) to generate cDNAs with the following conditions: 25°C for 10 min followed by 42°C for 60 min for cDNA synthesis, and 85°C for 5 min for termination.

### Real‐time PCR

Primers used in real time PCR for human and mouse Sgk1, Sgk1_v3 and 18S rRNA have been published (Raikwar et al. 2008, 2012). Other primer sequences were parvalbumin: mPvalb_F: AGTTGCAGGATGTCGATGACAGA; mPvalb_R: GGCCCACCATCTGGAAGAACTTTT and NaCa exchanger1: mNcx1_F: GCCGAGCATTTTACAGGATTCAAGC; mNcx1_R: GCCACAGTACCACAGTTCTCTAGAC. Quantitative real time PCR was performed in a Mx3000p Multiplex quantitative PCR system (Agilent Technologies) and dissociation curves were generated for all samples. The number of cycles, annealing temperature, and extension time were varied as appropriate for the abundance of transcripts, the melting temperature of primers, and the size of amplicons. All reactions were performed using 18S as a control for RNA loading and the results quantified using the delta delta Ct method (Raikwar et al. 2012).

### Statistical Analysis

Data are provided as arithmetic means ± standard error. All data were tested for significance with a Studentʹs *t*‐test (equal variances), Mann–Whitney (unequal variances) or one‐way ANOVA where applicable, using SigmaPlot^®^ 12 (San Jose, CA). In all figures statistical significance is indicated where *P* values <0.05 were considered statistically significant.

## Results

**Figure 1:**
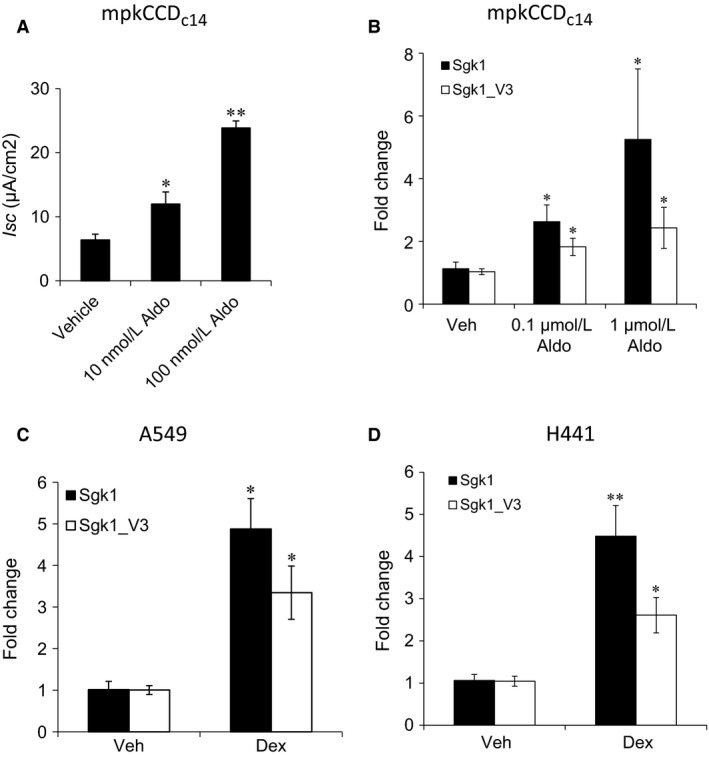
*Effect of corticosteroids on Na* ^*+*^ *transport and on sgk1_v3 expression*. (A) Effect of aldosterone for 4 h on short circuit current in mpkCCDc14 cells. Similar results were obtained for 24 h exposure from several independent experiments. (B) Effect of aldosterone for 24 h on sgk1 and sgk1_v3 mRNA expression in mpkCCDc14 cells. *n* = 8, mean ± SE from 2 exps. (C) Effect of 100 nm dexamethasone on Sgk1 and Sgk1_v3 mRNA expression in A549 cells for 4 h. Representative of several independent experiments. (D) Effect of 100 nm dexamethasone on Sgk1 and Sgk1_v3 mRNA expression in H441 cells for 2 h. *n* = 8, mean ± SE from 3 exps. *P < 0.05, **P < 0.01.

We have previously reported the primary structure and expression profile of the alternate 5ʹ variant sgk1 transcript, sgk1_v3 and showed that aldosterone and insulin increased sgk1_v3 expression in mpkCCD_c14_ cells. We also reported that its encoded protein sgk1_i3 is more stable compared to sgk1 and that it stimulates apical epithelial Na^+^ transport when expressed in FRT and in mpkCCD_c14_ cells (Raikwar et al. 2012). Sgk1 and Sgk1_i3 differ only in the NH_2_ terminus where 25AA in Sgk1 is replaced by 39 AA in Sgk1_i3. To further explore the basis for the corticosteroid stimulation of sgk1_v3 expression, we cultured mpkCCD_c14_ cells and demonstrated a dose‐dependent increase in short circuit current with aldosterone that correlated with a dose dependent increase in sgk1_v3 mRNA expression in mpkCCD_c14_ cells (Fig. 1A and B). We also demonstrate that dexamethasone stimulated an increase in Sgk1_v3 in the lung epithelial cell line, A549 and H441 (Fig. 1C, D). These results suggest that mineralocorticoids and glucocorticoids may increase the abundance of Sgk1_v3, similar to Sgk1, via a transcriptional regulatory element.

**Figure 2:**
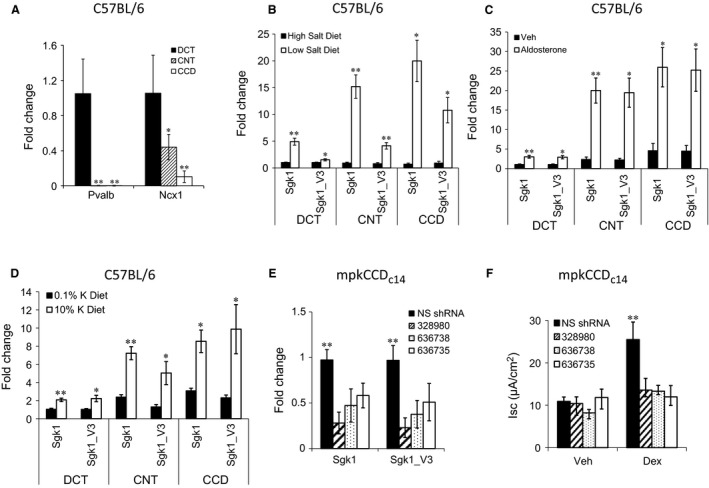
Effect of aldosterone and dietary Na^+^ and K^+^ manipulation on sgk1_v3 in vivo. (A) PCR amplification of tubule‐segment specific transcripts (Parvalbumin, PVA and Na^+^Ca^+^ exchanger, NCX1) in microdissected DCT, connecting tubule (CNT) and CCD. *n* = 6–7. *P < 0.05 ** P < 0.01. (B) Expression of Sgk1_v3 in microdissected tubule segments from mice placed on 0.13% NaCl vs 8% NaCl for 7 days. Induction of sgk1_v3 is seen in DCT, CNT and CCD with low Na^+^ diet. *n* = 6, mean ± SE, * *P* < 0.05 ** P < 0.01. (C) Expression of Sgk1_v3 in microdissected tubule segments from mice administered aldosterone or vehicle IP for 2 hrs. Induction of sgk1_v3 is seen in CNT and CCD with aldosterone. *n* = 5, mean ± SE, * *P* < 0.05 ** P < 0.01. (D) Expression of Sgk1_v3 in microdissected tubule segments from mice placed on 0.1% K^+^ vs 10% K^+^ for 7 days. Induction of sgk1_v3 is seen in CNT and CCD with high K^+^ diet. *n* = 6, mean ± SE, * *P* < 0.05 ** P < 0.01. (E) Expression of sgk1 and sgk1_v3 mRNA in mpkCCDc14 cells selected with puromycin after transduction with non‐specific (NS) lentiviral shRNA or sgk1 lentiviral shRNAs 32890, 63678 or 636735. Reduced expression of sgk1 and sgk1_v3 is seen in sgk1 shRNA transduced cells *n* = 7, mean ± SD, ** P < 0.01. (F) Effect of dexamethasone for 24 h on short circuit current (Isc) in stable shRNA transduced mpkCCDc14 cells. Dexamethasone stimulates *Isc* in NS shRNA cells but not in sgk1 shRNA cells *n* = 3, mean ± SD, ** *P* < 0.01.

To determine the physiological importance of regulated expression of Sgk1_v3 in the distal nephron, we first performed additional experiments in mice, testing separately the effects of Na^+^ deprivation, the effects of dietary K^+^ excess and the effects of short‐term aldosterone infusion on different distal nephron segments. We microdissected DCT, CNT, and CCD from mouse kidney cortex as previously reported (Itani et al. 2005). To verify the identity of the nephron segments we tested the expression of parvalbumin and the Na^+^Ca^2+^ exchanger NCX by qPCR and showed that nephron segments identified as DCT showed parvalbumin expression while CNT and CCD segments were negative as has been reported (Câmpean et al. 2001). We identified NCX1 in all three segments with highest expression in the DCT, less expression in the CNT and very low expression in the CCD (Fig. 2A). NCX1 is known to be expressed in the DCT2 and in CNT although prior literature has been mixed on the presence of NCX1 in the CCD (Loffing et al. 2001; Biner et al. 2002). Each of the three ENaC subunits were also identified in all the distal nephron segments (data not shown).

We then performed qPCR in nephron segment mRNA and show that both sgk1 and sgk1_v3 were increased in each of the segments after 1 week period of dietary Na^+^ modification (Fig. 2B). The increase in sgk1 and sgk1_v3 is consistent with an increase in angiotensin II‐mediated aldosterone secretion leading to an increase in sgk1 gene transcription as has been described for glucocorticoid‐stimulated sgkl (Webster et al. 1993; Itani et al. 2002). We also tested the effect of a short‐term exposure to aldosterone on sgk1 and sgk1_v3 expression and found similar increases in both sgk1 and sgk1_v3 (Fig. 2C). We then examined the effect of high K^+^ versus low K^+^ diet on sgk1_v3 expression as hyperkalemia is also a stimulus for aldosterone secretion. We show that a high K^+^ diet stimulates sgk1 and sgk1_v3 expression consistent with an effect of aldosterone on sgk1 gene transcription (Fig. 2D). Although the magnitude of the effect cannot be directly compared, there appeared to be a larger induction of sgk1 and sgk1_v3 expression in the CNT and CCD compared to the DCT which may reflect the increased dependence of sgk1 on MR in these nephron segments (Verrey et al. 2003; Czogalla et al. 2016).

We performed sgk1 knockdown experiments using lentiviral expression of Sgk1 shRNA targeted to a sequence common to both sgk1 and sgk1_i3. We demonstrate efficient knockdown of both transcripts and showed that this knockdown was sufficient to reduce dexamethasone stimulated Na^+^ transport with no effect on basal transport, indicating that sgk1 is necessary for the corticosteroid‐mediated increase in Na^+^ transport at least in cultured CCD cells (Fig. 2E and F). Given the limited region of sequence diversity between Sgk1 and sgk1_v3 we were unable to design and selectively knockdown sgk1_v3 (data not shown).

**Figure 3:**
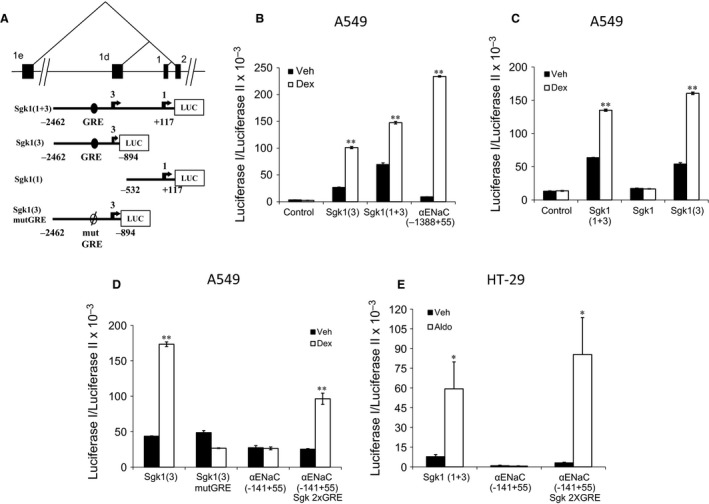
Identification of cis‐elements required for corticosteroid‐mediated Sgk1_v3 expression. (A) Schematic showing proximal exons (1e, 1d, 1 and 2) and intervening regulatory regions for human Sgk1 gene as well as the sequence present in each of the Sgk1 luciferase (LUC) constructs. (B), (C) and (D) A549 cells transfected with indicated constructs and treated with 100 nmol/L dexamethasone, or vehicle for 24 h. *n* = 3, mean ± SE ** P < 0.01. Constructs that do not contain exon 1d and sequence 5ʹ are not dexamethasone responsive. Selective mutation of the GRE abolishes dexamethasone responsiveness. (E) HT‐29 cells cotransfected with luciferase constructs and rat MR or empty plasmid and treated with 100 nmol/L aldosterone or vehicle for 24 h. Aldosterone increases luciferase activity via the GRE in the Sgk1_v3 5ʹ regulatory region. *n* = 3, mean ± SE * P < 0.05. This GRE is sufficient to confer aldosterone responsiveness to a heterologous construct.

Sgk1_v3 is a 5ʹ variant alternate transcript of the Sgk1 gene that has a different transcription start site and a different 5ʹ proximal regulatory region from Sgk1 (Fig. 3A). Others and we have demonstrated that glucocorticoid regulation of Sgk1 is mediated via a GRE in the 5ʹ flanking region of Sgk1 (Webster et al. 1993; Itani et al. 2002). A careful analysis of the 5ʹ flanking region of Sgk1 demonstrated that the GRE that was reported to be 1152 bp upstream of the Sgk1 transcription start site is also 319 bp upstream of the Sgk1_v3 transcription start site since the first exon for Sgk1_v3 (exon 1d) is 834 bp upstream of the first exon for Sgk1 (exon 1). To determine if this GRE functioned to regulate Sgk1 and Sgk1_v3 we cloned this fragment upstream of a luciferase reporter gene and then isolated the 5ʹ flanking region for both exons. A ~2600 bp construct, Sgk1(1 + 3), that included the GRE, the complete exon 1d and the transcription start site for exon 1 conferred glucocorticoid‐responsiveness to the luciferase reporter in A549 cells (Fig. 3B). A ~1600 bp construct, Sgk1(3), that included the GRE and the transcription start site for exon 1d but not downstream sequence was also glucocorticoid‐responsive indicating that this region was sufficient to stimulate transcription of Sgk1_v3 (Fig. 3B). In comparison, a ~500 bp region upstream of exon 1 that excluded exon 1d and sequences 5ʹ to it was not glucocorticoid responsive (Sgk1) indicating that exon 1d or sequences 5ʹ to it are necessary for the glucocorticoid response of the Sgk1 promoter construct (Fig. 3C). To confirm that the GRE mediated the GC responsiveness for exon 1d, we selectively mutated the element from −1234 to −1249 within the promoter reporter construct (Sgk1(3)mutGRE) and demonstrated loss of GC responsiveness (Fig. 3D). To verify that the GRE was sufficient for the GC response we transferred two copies of this GRE to a promoter reporter construct containing the 5ʹ flanking region of αENaC (aENaC(−141 + 55)Sgk2xGRE). We demonstrate that the αENaC construct containing the Sgk1_v3 GRE becomes GC responsive (Fig. 3D).

Finally, to demonstrate that this GRE could also respond to aldosterone we tested this promoter‐reporter construct in HT‐29 cells, a colonic epithelial cell line where the MR is not expressed (Mick et al. 2001). We demonstrate that when MR is coexpressed, aldosterone stimulated luciferase expression from the Sgk1 luciferase construct and from a heterologous αENaC promoter that also included the Sgk1 GRE (Fig. 3E). These results confirm that glucocorticoid and mineralocorticoid regulation of Sgk1_v3 and Sgk1 expression is mediated via a shared hormone response element (HRE) in the 5ʹ regulatory region of Sgk1. The results also verify that a low Na^+^ diet or a high K^+^ diet increase Sgk1 and Sgk1_v3 expression similar to aldosterone, effects that are probably mediated via aldosterone‐regulated Sgk1 gene transcription.

## Discussion

Sgk1 is a highly regulated kinase that is expressed in the DCT, CNT and CCD, nephron segments that constitute the aldosterone‐sensitive distal nephron. Aldosterone is one of the principal regulators of several transport pathways that directly or indirectly regulate Na^+^ transport in these nephron segments. These include the electroneutral thiazide‐sensitive NCC in the DCT, the electrogenic amiloride‐sensitive ENaC in DCT2, CNT and the principal cells of the CCD, and pendrin and the Na^+^‐driven Cl^‐^/HCO3^+^ exchanger NDCBE in *β*‐intercalated cells of the CCD which together subserve a thiazide‐sensitive electroneutral Na^+^ reabsorptive pathway in the CCD (Pao 2012; Eladari et al. 2014; Wall 2016). Sgk1 is known to regulate ENaC and NCC but it is not known if Sgk1 has any effects on pendrin or NDCBE. Sgk1 has two paralogs, sgk2 and sgk3, that probably arose as a gene duplication event, and these proteins are also capable of stimulating epithelial Na^+^ transport, at least in a Xenopus oocyte expression system (Friedrich et al. 2003; Pao et al. 2007). Sgk2 is not expressed in the DCT and at very low levels in the CCD and sgk2 knockout mice do not have any evidence of salt wasting suggesting that sgk2 does not interact with ENaC in the distal nephron in vivo (Pao 2012). Likewise, sgk3 is expressed at very low levels in the distal nephron and sgk3 knockout mice show impaired hair follicle development but no renal phenotype (McCormick et al. 2004). Sgk1 also has several alternate splice forms, Sgk1_v2 and Sgk1_v3 that lead to N‐terminal variants Sgk1_i2 and Sgk1_i3. We previously showed that Sgk1_v3 is an aldosterone‐regulated transcript and that Sgk1_i3, like Sgk1, stimulates ENaC mediated Na^+^ transport (Raikwar et al. 2012).

Here, we show sgk1_v3 is not only aldosterone‐regulated in an established collecting duct cell line, but that it is also glucocorticoid‐regulated in airway and alveolar epithelial cell lines. The aldosterone effect is likely to be physiologically relevant since aldosterone infusion and maneuvers that increase endogenous aldosterone secretion such as dietary Na^+^ restriction and K^+^ loading increases Sgk1_v3 expression in the DCT, CNT, and CCD *in vivo*. We verified that the effect of corticosteroids, dexamethasone and aldosterone on Sgk1_v3 expression is mediated via the HRE that had been previously shown to regulate Sgk1 in human airway epithelia (Itani et al. 2002). However we do not know if the increase in sgk1_v3 is physiologically relevant as we were unable to selectively knockdown sgk1_v3.

The HRE in the Sgk1 gene is an imperfect palindromic bipartite sequence within the 5ʹ regulatory region that is identical to that seen in several non‐human primates. Interestingly, the transcription start site is upstream of and incorporates this GRE within the 5ʹ transcribed but untranslated region for several other primates including the *macaca nemestrina* (pig tailed macaque), *chlorocebus sabaeus* (green monkey) and *microcebus murinus* (gray mouse lemur). The primate HRE differs from rat and mouse sgk1 HRE, ACCACAnnnTGTTCT by 2 nucleotides. We have demonstrated that sgk1_v3 is conserved in rodents and like sgk1 that it is regulated by glucocorticoids in human airway epithelial cells (A549) and mouse collecting duct epithelial cells (mpkCCDc14). It is not uncommon for a 5ʹ regulatory element to regulate multiple transcripts of the same gene even when the transcription start sites and proximal promoters are different. While exon 1d and exon 1, the initiating exons for Sgk1_v3 and Sgk1 are only ~700 bp apart in *homo sapiens*, the transcription start site for exon 1a, the initiating for Sgk1_v2 is a ~135 kb away. Since Sgk1_v2 is not corticosteroid regulated, a transcriptional insulator element is likely to be present upstream of the Sgk1 HRE shielding Sgk1_v2 and possibly other upstream genes from the influence of this HRE.

In conclusion, we demonstrate that glucocorticoids and aldosterone regulate an abundant 5ʹ variant alternate transcript of Sgk1, Sgk1_v3 in multiple epithelial cell lines and physiologically relevant stimuli such as aldosterone, low Na^+^ diet and high K^+^ diet regulate Sgk1_v3 in vivo. We identified a shared 5ʹ flanking HRE as the transcriptional basis for corticosteroid‐stimulated Sgk1_v3 and sgkl expression.

## Conflict of Interest

None declared.
